# Physical Activity Measurement Reactivity Among Midlife Adults With Elevated Risk for Cardiovascular Disease: Protocol for Coordinated Analyses Across Six Studies

**DOI:** 10.2196/67438

**Published:** 2025-04-23

**Authors:** Kiri Baga, Gabrielle M Salvatore, Iris Bercovitz, Amanda L Folk, Ria Singh, Laura M König, Meghan L Butryn, Jacqueline A Mogle, Danielle Arigo

**Affiliations:** 1 Department of Psychology Rowan University Glassboro, NJ United States; 2 Faculty of Psychology University of Vienna Vienna Austria; 3 Weight, Eating, and Lifestyle Science (WELL) Center Department of Psychological and Brain Sciences Drexel University Philadelphia, PA United States; 4 RTI Health Solutions Durham, NC United States; 5 Department of Family Medicine Rowan-Virtua School of Osteopathic Medicine Stratford, NJ United States; 6 Department of Biomedical Sciences Cooper Medical School of Rowan University Camden, NJ United States

**Keywords:** physical activity, measurement reactivity, gender difference, cardiovascular risk, intensive assessment, midlife, research participation effects

## Abstract

**Background:**

Cardiovascular disease (CVD) remains the leading cause of death in the United States, and adults aged 40-60 years with specific health conditions are at particularly elevated risk for developing CVD. Physical activity (PA) is a key cardioprotective behavior and many interventions exist to promote PA in this group. Effective promotion requires accurate assessment of PA behavior; as PA is often estimated by averaging across multiple days, a threat to accurate assessment is *measurement reactivity*, or an atypical increase in PA behavior at the start of measurement periods that may bias conclusions. Evidence for PA measurement reactivity is equivocal, though concern has resulted in recommendations to add or drop PA measurement days from inclusion, which may introduce undue burden on participants. At present, the extent of PA measurement reactivity and the behaviors most likely to be affected (eg, steps vs minutes of exercise) among those at risk for CVD are unclear, as are participant characteristics such as gender and study expectations (eg, intervention vs observation only) that may contribute to differences in these patterns.

**Objective:**

The goal of this study is to improve on the current understanding of the extent of PA measurement reactivity and potential moderators among US adults aged 40-60 years with CVD risk factors.

**Methods:**

To achieve this goal, we will conduct coordinated multilevel analyses across 6 studies. Data are from nationally representative, publicly available datasets (observation only: 2 studies) and baseline weeks of observation from behavioral weight loss clinical trials (4 studies), all collected in the United States. The publicly available datasets National Health and Nutrition Examination Survey (NHANES; 2013-2014) and the Midlife in the United States (MIDUS) Study (2004-2009; total n=1385) were used, which are available from the Inter-university Consortium for Political and Social Research website. Behavioral weight loss trials were conducted by the Drexel University Weight Eating and Lifestyle (WELL) Center (2011-2023; total n=444), in person or remotely via Zoom. Relevant data from each study were extracted for adults aged 40-60 years who have ≥1 risk factor for CVD (total n=1832; 11,707 total days of PA measurement with 6-7 days per person). Changes in PA behavior across the measurement period will be examined at the day level, using 2-level multilevel models (days nested within persons) and cross-level interactions (for moderation effects).

**Results:**

This project was funded in August 2022 and received supplementary funding in September 2023. Dataset acquisition and data cleaning were completed in October 2024. Analyses are expected to be completed in April 2025, and findings are anticipated to be shared in July 2025.

**Conclusions:**

Results from this coordinated analysis project will provide the first large-scale estimation of the extent of PA measurement reactivity in an at-risk group. Findings will inform best practices for mitigating potential measurement reactivity in multiday assessments of PA behavior.

**International Registered Report Identifier (IRRID):**

DERR1-10.2196/67438

## Introduction

Cardiovascular disease (CVD) remains the leading cause of death in the United States, and risk for CVD meaningfully increases during midlife (ages 40-60 years) [1,2]. Increased risk for CVD during midlife is due to universal aging processes and associated conditions such as obesity and hypertension, as well as to sex-specific metabolic and hormonal changes (eg, the onset of menopause), which result in distinct risk patterns for women and men [2-6]. Engaging in regular physical activity (PA) during midlife can significantly reduce the risk of CVD for both women and men [7,8]. Unfortunately, low PA engagement among midlife adults is common, which exacerbates their CVD risk and contributes to their substantial health care costs [4,9]. This problem persists despite decades of efforts to promote PA that include high participation rates among midlife adults [10,11], and when PA promotion is effective, the benefits of participation are short-lived [12]. This applies to the many PA interventions that are specifically tailored for midlife adults, including those that are gender-specific (ie, enroll only men or women or focus on sex- or gender-specific content, such as PA in the context of gender roles, effects of PA on menopause symptoms, or risk for prostate cancer) [13-18]. Evidence consistently shows that very few midlife adults—particularly those with CVD risk conditions such as obesity, hypertension, or type 2 diabetes—achieve PA levels that would confer significant protection against CVD [19-21]. Thus, additional work is needed to effectively promote PA to reduce CVD risk among midlife adults.

Critically, however, our understanding of current PA engagement and the benefits of PA for reducing CVD risk among midlife adults relies on accurate PA measurements in this at-risk population. Assessment of PA using research-grade or commercially available devices (eg, Fitbit) provides more accurate estimates of PA engagement than methods such as self-report [22]. To ensure accurate estimates by accounting for normal daily variability in PA engagement [23], monitoring periods typically last 7 days, and observed PA behavior (eg, steps per day, minutes of moderate-to-vigorous-intensity activity) is averaged across these days to generate an overall estimate of PA engagement [24]. Evidence across the multidisciplinary field of PA promotion highlights the potential for bias in the assessment of PA using monitoring technology; one source of this bias is *measurement reactivity*, or change in PA behavior due to the introduction of its measurement [17,21,25,26]. Introducing PA monitoring via an external device during participation in a research study is thought to increase the salience of both immediate PA engagement and the notion that someone else is watching [27,28]. This increased awareness can lead to significantly more PA engagement early in the monitoring period than is typical or sustainable. Specifically, PA measurement reactivity typically manifests as greater PA at the start of observation periods, relative to subsequent days (eg, negative linear slope across days, or higher on day 1 or days 1-2) [25]. When estimates are calculated as averages across days, initial elevation could skew these estimates to show greater engagement than is typical or sustainable [25]. The resulting inflation of PA estimates could mask the effects of PA interventions, as preintervention starting points could be overestimated [17] (among other confounds), leading to incorrect conclusions about PA engagement among at-risk groups such as adults in midlife.

To fully understand the value of promoting PA to reduce CVD risk among adults in midlife with existing risk factors, it is critical to ensure the accuracy of PA measurements in this population. As noted, if measurement reactivity effects are substantial, the resulting biased estimates of PA in at-risk groups such as midlife adults could lead to incorrect conclusions and next steps for PA promotion. PA measurement reactivity has been observed in a subset of studies to date [25,29-31], and some PA researchers consider reactivity a critical source of bias that warrants increased attention and mitigating actions, such as requiring extra days of observation to get used to the PA monitor or removing the first 1-2 days of PA observation from analyses [30]. The evidence is equivocal, however, with some studies showing no reactivity patterns or patterns that are not clinically significant (and thus, would not meaningfully affect overall PA estimates) [25,28]. As a result, there is considerable debate about the need for such drastic measures, or even the need for ongoing attention to this phenomenon. PA recordings on these days represent actual PA engagement, even if the level of engagement is not “typical,” and adding assessment days increases participant burden [32]. Consequently, the extent of PA measurement reactivity and best practices for handling PA data to address the potential for it to affect estimates have yet to be identified.

Existing evidence is also limited in three key ways. First, studies of PA measurement reactivity have often focused on children or healthy, younger adults. Because these populations generally are more active than midlife adults with elevated risk for CVD (or similar at-risk groups) [19,33], they may respond differently to the introduction of PA monitoring. Second, there is little consistency between existing studies with respect to definitions of reactivity or which PA outcomes are affected; outcomes of interest have included steps per day, light PA, moderate-to-vigorous PA (MVPA), and sedentary time [29,30,33], which have distinct implications for CVD risk that differ by gender [34,35]. In addition, some work has defined reactivity as an overall linear decrease in PA across days of observation [25,36], whereas others have examined direct comparisons between day 1 and subsequent days (days 2, 3, 4, or combinations) [29,37]. Third, there has been little attention to the contexts where PA measurement reactivity is a meaningful problem, leading to inconsistent and often drastic recommendations for addressing it. As noted, these include adding observation days, which increases participant burden, and removing the first 1-2 days of observation, which reduces the precision of estimates (or both of these steps) [25,27,30,38]. Although there is some convergence in PA estimates between commercially available wearable devices and research-grade monitors [39], differences in the location of wear (eg, wrist vs hip) and the extent to which a device offers PA feedback (eg, via a digital display of accumulated steps per day in real time) could influence reactivity by differentially increasing the salience of PA [31]. Assessment of PA prior to the start of an intervention (vs in studies that use observation alone) may also affect reactivity via differences in participants’ anticipation of future increases in their PA [30].

In addition, individual differences in mental health symptoms, physical health characteristics, and psychosocial processes are known determinants of PA engagement and thus may influence reactivity patterns [39]. For example, depressive symptoms, number of cardiovascular risk factors, and BMI are negatively associated with PA engagement [40,41] and, as a result, may limit reactivity. In contrast, tendencies toward self-evaluation relative to others and increased motivation for health behaviors may have the opposite effects [40,42]. Specifically, women and those who have stronger tendencies to make and value social comparisons (ie, self-evaluations of one’s traits or behaviors compared to others [43]) may show greater measurement reactivity responses, as these individuals may be particularly interested in managing others’ perceptions of them (such as researchers) [43-46]. Higher (vs lower) levels of PA motivation and more (vs fewer) past attempts at PA behavior change may also be associated with stronger reactivity responses, as these may indicate greater focus on PA overall [47]. Greater attention to individual differences and contexts, rather than treating PA measurement reactivity as universally problematic versus not (as it is currently), could identify optimal and tailored targets for mitigating reactivity in future studies.

Thus, the role of PA measurement reactivity in PA promotion for at-risk groups remains unclear, and no existing study has examined the extent of reactivity based on research design, study procedures, and individual participant characteristics. For this study, we will capitalize on the availability of existing data resources with a multilevel coordinated analysis across 6 studies (including 2 nationally representative observational studies and 4 intervention trials) to achieve the following aims:

1. To characterize midlife adults’ PA measurement reactivity in each dataset. We will determine whether daily PA engagement meaningfully changes over 6-7 days of observation, across available PA outcomes. Available data include monitor-independent measurement summary (MIMS) units, activity counts, and steps per day.
2. To determine whether the presence or extent of measurement reactivity differs based on demographic, medical, or psychological characteristics. We hypothesize that reactivity will be weaker among adults with higher (vs lower) BMIs, numbers of CVD risk factors, and depressive symptoms. Conversely, we hypothesize that reactivity effects will be stronger among women, adults with higher (vs lower) PA motivation and social comparison tendencies, and those with more previous attempts to increase PA or lose weight. We will further examine whether gender moderates the effects of other individual difference characteristics on PA engagement over 6-7 days by including the interaction effect between gender and other predictors.
3. To determine whether the presence or extent of measurement reactivity differs based on study characteristics. We hypothesize that reactivity effects will be stronger in studies that use observation only (as there is no expectation of later improvement), commercially available devices (which tend to be visible throughout the day), wrist wear (based on both visibility and sensitivity to movement [31,48]), and devices that offer feedback in real time (as they provide additional information that can prompt a behavioral response [49]). We will further examine whether gender moderates the effects of study characteristics on PA engagement over 6-7 days by including the interaction effect between gender and other predictors.

## Methods

### Overview

Using a coordinated analysis approach [50], we will model differences in PA engagement across days of observation using data from each of the 6 studies. These include 2 observational studies that have publicly available datasets and 4 clinical trial datasets. All data were collected between January 2005 and January 2023. Procedures included 6-7 days of intensive PA assessment using a research-grade or commercially available device. Analysis of available datasets leverages existing resources to address questions beyond those originally intended; this limits cost and eliminates additional participant burden while offering insights into important phenomena [51]. This approach is uniquely suited for the present study, as the concern about PA measurement reactivity is that it appears any time PA monitoring devices are introduced. If this concern is warranted, evidence of measurement reactivity should be observed in studies that were not specifically designed to test this phenomenon (eg, studies for which PA was assessed for other purposes and data are already available). The datasets used for this set of secondary analyses were selected for their accessibility to the research team, representation of a large number and range of individuals in the target population, and heterogeneity in study designs and monitoring PA devices used. Together, these datasets will enable examination of reactivity patterns following the introduction of PA measurement devices in multiple people and contexts.

### Observational Studies

These include samples from the National Health and Nutrition Examination Survey (NHANES) [52] and the Midlife in the United States (MIDUS) [1] Study (combined n=1385). NHANES is a national, longitudinal program of research focused on understanding various aspects of health among adults and children in the United States, and data are publicly available. We will use data from the most recent wave of collection that included PA monitoring (2013-2014; 7 days of assessment with the ActiGraph GT3X). MIDUS is a longitudinal investigation of life circumstances and health outcomes among adults aged 25-75 years. The present analyses use data from the MIDUS Biomarker Project (2004-2009), which included PA monitoring (6 days of assessment with the Actiwatch-64).

### Randomized Clinical Trials

These studies were tests of improvements to standard behavioral weight loss treatment (4 studies, combined n=444). Each study used a 7-day pretreatment observation window, which will be used to examine PA measurement reactivity. Project ENACT (NCT01858714) tested the effects of an enhanced focus on the food environment and the use of acceptance and commitment skills to support behavior change on long-term weight loss [53]. The collection of the data to be used in the planned analyses began in 2011 and finished in 2013. Project IMPACT (NCT02363 010) tested an increased emphasis on PA and acceptance and commitment skills to support behavior change on long-term weight loss [54]. Collection of the data to be used in the planned analyses began in 2014 and finished in 2016. The last 2 studies examined the added benefits of sharing PA self-monitoring data, either with coaches (Project FitLink Pilot, 2018-2019; NCT03337139 [55]) or with coaches, a designated member of the participant’s existing social network (friend or family member), and other participants (Project FitLink Full, 2021-2023; NCT05180448 [56]). ENACT and IMPACT used the same research-grade accelerometer (Actigraph GT3X), worn on the hip. FitLink Full used a commercially available wearable PA monitor (Fitbit Inspire 2), worn on the wrist, and FitLink Pilot used both the research-grade hip-worn accelerometer and a commercially available wrist-worn monitor (Fitbit Flex; Table 1). The commercial device was worn from the baseline period through treatment; the research-grade device was added at a 3-month assessment (ie, at randomization to condition after a uniform treatment period). ENACT, IMPACT, and FitLink Pilot were conducted in person in a large city in the northeastern United States, whereas FitLink Full was conducted remotely using national recruitment and enrollment.

**Table 1:** Physical activity measurement by study.

Study		Device	Duration	Outcomes
**Observational studies**				
	NHANES^a^ (n=1208)	ActiGraph GT3X (wrist-worn)	7 days	Monitor-independent movement summary units
	MIDUS^b^ (n=177)	Actiwatch-64 (wrist-worn)	6 days	Total activity count units
**Randomized clinical trials (intervention programs)**				
	ENACT (n=143)	ActiGraph GT3X+ (hip-worn)	7 days	Steps per day
	IMPACT (n=140)	ActiGraph GT3X+ (hip-worn)	7 days	Steps per day
	FitLink Pilot (n=31)	Fitbit and ActiGraph GT3X (hip-worn; week 13)	7 days	Steps per day
	FitLink Full (n=130)	Fitbit	7 days	Steps per day

^a^NHANES: National Health and Nutrition Examination Survey.

^b^MIDUS: Midlife in the United States.

### Participants

Eligible participants are adults in midlife (aged 40-60 years) with ≥1 risk factors for CVD who completed the relevant PA assessment period (see below for additional details about inclusion in data analyses). CVD risk factors include prediabetes, type 2 diabetes, hypertension, high cholesterol, obesity, and current smoking. As noted, observational studies (n=1385) recruited nationally in the United States; clinical trials (n=444) were behavioral weight loss studies completed in the northeastern United States, one of which was conducted remotely. Participant demographics are listed in Table 2.

**Table 2:** Participant demographics across 6 studies.

					NHANES^a^ (n=1208)	MIDUS^b^ (n=177)	ENACT (n=143)	IMPACT (n=140)	FitLink Pilot (n=31)	FitLink Full (n=130)
Age (years), mean (SD)					49.9 (6.0)	50.9 (5.8)	52.3 (5.6)	52.9 (5.0)	50.9 (6.1)	51.5 (5.7)
**Gender,** **n (%)**										
				Women	628 (52.0)	105 (59.3)	117 (81.8)	116 (82.9)	28 (90.3)	109 (83.8)
				Men	580 (48.0)	72 (40.7)	26 (18.2)	24 (17.1)	3 (9.7)	21 (16.2)
BMI (kg/m^2^), mean (SD)					31.2 (7.4)	32.0 (7.2)	36.3 (4.5)	35.5 (4.4)	36.3 (4.5)	36.5 (4.8)
**BMI category,** **n (%)**										
				<18.5 kg/m^2^	9 (0.8)	1 (0.6)	0 (0.0)	0 (0.0)	0 (0.0)	0 (0.0)
				18.5-25 kg/m^2^	224 (18.6)	26 (14.7)	0 (0.0)	0 (0.0)	0 (0.0)	0 (0.0)
				25-30 kg/m^2^	321 (26.6)	43 (24.3)	7 (4.9)	6 (4.4)	0 (0.0)	4 (3.1)
				>30 kg/m^2^	653 (54.1)	107 (60.4)	136 (95.1)	131 (95.6)	31 (100.0)	126 (96.9)
**Race^c^, n (%)**										
			American Indian or Native Alaskan		—^d^	0 (0.0)	1 (0.7)	0 (0.0)	1 (3.2)	0 (0.0)
			Asian		115 (9.8)	2 (1.1)	1 (0.7)	1 (0.7)	0 (0.0)	3 (2.3)
			Native Hawaiian or Other Pacific Islander		—	0 (0.0)	1 (0.7)	0 (0.0)	0 (0.0)	0 (0.0)
			Black or African American		273 (23.4)	50 (28.4)	51 (35.7)	35 (25.0)	12 (38.7)	17 (13.1)
			White		486 (41.6)	103 (58.5)	84 (58.7)	98 (70.0)	14 (45.2)	107 (82.3)
			Other or mixed race		32 (2.7)	21 (11.9)	5 (3.5)	6 (4.3)	4 (12.9)	3 (2.3)
**Ethnicity^c^, n (%)**										
	Hispanic or Latino				262 (22.4)	0 (0.0)	8 (5.6)	5 (3.6)	2 (6.4)	10 (7.7)
	Not Hispanic or Latino/a				906 (77.6)	177 (100.0)	134 (94.4)	135 (96.4)	29 (93.6)	120 (92.3)
**Marital status,** **n (%)**										
		Married			685 (58.6)	100 (56.5)	84 (59.2)	91 (65.0)	15 (48.4)	90 (69.2)
		Widowed			28 (2.4)	4 (2.3)	2 (1.4)	2 (1.4)	1 (3.2)	1 (0.8)
		Divorced			198 (17.0)	32 (18.1)	22 (15.5)	21 (15.0)	4 (12.9)	8 (6.2)
		Separated			52 (4.4)	9 (5.1)	3 (2.1)	5 (3.6)	3 (9.7)	—
		Never married			143 (12.2)	32 (18.1)	31 (21.8)	21 (15.0)	8 (25.8)	—
		Single			—	—	—	—	—	20 (15.4)
		Cohabitating			61 (5.2)	—	—	—	—	9 (6.9)
		Not cohabitating			—	13 (16.9)	—	—	—	2 (1.5)
**Income^e^ (US $), n (%)**										
		$0-$25,000			294 (25.5)	63 (36.6)	9 (6.4)	4 (2.9)	1 (3.2)	—
		$25,000-$50,000			210 (18.2)	53 (30.8)	21 (14.9)	13 (9.5)	4 (12.9)	—
		$45,000-$55,000			94 (8.2)	—	—	—	—	—
		$50,000-$75,000			126 (10.9)	27 (15.7)	31 (22.0)	15 (11.0)	7 (22.6)	—
		$75,000-$100,000			113 (9.8)	14 (8.1)	24 (17.0)	23 (16.8)	6 (19.4)	—
		>$100,000			251 (21.8)	8 (4.6)	13 (9.2)	20 (14.6)	2 (6.4)	—
		$125,000-$150,000			—	2 (1.2)	18 (12.8)	17 (12.4)	6 (19.4)	—
		$150,000-$175,000			—	2 (1.2)	8 (5.7)	18 (13.1)	0 (0.0)	—
		$175,000-$200,000			—	0 (0.0)	5 (3.6)	13 (9.5)	2 (6.4)	—
		>$200,000			—	3 (1.7)	12 (8.5)	14 (10.2)	3 (9.7)	—
**Education,** **n (%)**										
		Less than 9th grade			80 (6.8)	2 (1.1)	0 (0.0)	0 (0.0)	0 (0.0)	—
		Partial high school			156 (13.4)	10 (5.6)	1 (0.7)	0 (0.0)	0 (0.0)	—
		High school or GED^f^			280 (24.0)	35 (19.8)	13 (9.1)	5 (4.3)	1 (3.2)	—
		Associate’s degree, technical, or partial college			373 (31.9)	52 (43.5)	22 (15.4)	19 (16.4)	4 (12.9)	—
		Bachelor’s degree			279 (23.9)	44 (24.9)	52 (36.4)	41 (35.3)	11 (35.5)	—
		Graduate or professional degree			—	34 (19.2)	55 (38.5)	51 (44.0)	15 (48.4)	—

^a^NHANES: National Health and Nutrition Examination Survey.

^b^MIDUS: Midlife in the United States.

^c^Race and ethnicity questions were combined for NHANES.

^d^Not available.

^e^Income was based on household, not individual, and had different cutoff values.

^f^GED: General Education Development.

### Procedures

Datasets were chosen for the availability of consecutive days of PA measurement captured immediately following the introduction of commercial and research-grade accelerometers. PA data were recorded using research-grade or commercially available devices (see Table 1). Participant demographics and individual difference characteristics were collected using questionnaires or structured interviews. Specific details about these measures are described in detail below.

### Measures

#### Demographics

Participants’ basic demographics were collected via self-report. These data included age, gender, racial and ethnic identity, marital status, income, and education (see Table 2). NHANES collected these data during structured, in-person interviews with participants. The remaining studies captured these data using electronic or paper questionnaires.

#### Physical Activity

Included studies captured 6-7 days of PA data using different PA monitoring devices and will enable examination of device-specific effects. ActiGraph devices were used for observational studies and 3 (of 4) clinical trials. These devices were wrist-worn and hip-worn ActiGraph models for observational studies and clinical trials, respectively; remaining clinical trials used commercially available Fitbit devices (see Table 1). PA outcomes were daily PA summary metrics or steps per day. Observational studies used MIMS units [57] and total activity counts; clinical trial datasets provided steps per day (also detailed in Table 1). Steps per day captured using Fitbit at baseline and both a Fitbit and ActiGraph at 3 months into treatment during the FitLink Pilot will allow for comparison of potential recurrence of reactivity patterns after the onset of behavioral weight loss treatment.

#### Individual Difference Measures

Measures of individual differences are shown in Table 3.

*CVD risk conditions* include prediabetes or type 2 diabetes, hypertension, high cholesterol, obesity, or current smoking. Participants were prompted to report whether they had a physician diagnosis of prediabetes, type 2 diabetes, hypertension, and high cholesterol. BMI ≥30 kg/m2 will be used to indicate obesity and will be calculated based on measured height and weight. Table 4 summarizes the risk factors assessed in each study. Participants with pre-existing CVD will be excluded from analyses, including those who self-report a history of heart disease, heart failure, stroke, or heart attack. We will calculate the proportion of CVD risk factors endorsed out of the risk factors assessed to determine an overall risk percentage.

*Depressive symptoms* were measured in each study using validated self-report measures, including the Patient Health Questionnaire (PHQ-9) [58], the Center for Epidemiologic Studies Depression Scale (CES-D) [59], and the Beck Depression Inventory [60]. Total scores for these measures range from 0 to 27, 0 to 60, and 0 to 63, respectively, with higher scores indicating more severe depressive symptoms. Depressive symptoms were captured for the FitLink Pilot using the Weight and Lifestyle Inventory (WALI [61]), which prompts participants to report experience of depressed mood or anhedonia during the past month.

*Social comparison* will be examined for 3 datasets (ie, MIDUS, ENACT, and FitLink Full). MIDUS captured health-related social comparison experiences; participants were prompted to indicate their perception of their level of risk for a heart attack compared to others (ie, higher, lower, or the same) and the degree of this difference (eg, a lot higher, somewhat higher, or only a little higher). Social comparison experiences for clinical trial participants were measured using the Iowa-Netherlands Comparison Orientation Measure (INCOM) [43]. This measure assesses respondents’ perceptions of their own tendency to make social comparisons, generally and tendencies toward upward (ie, better off) and downward (ie, worse off) comparison targets. Higher total scores indicate stronger tendencies toward social comparison.

*Motivation to be physically active* was assessed for 3 datasets (clinical trials; ie, ENACT, IMPACT, FitLink Pilot) using an adapted version of the Treatment Self-Regulation Questionnaire (TSRQ [62]). This measure assesses different types of motivation for changing health behaviors by capturing the extent to which respondents endorse particular reasons for changing their health behavior on a scale (from 1=not at all true to 7=very true). Specific TSRQ item scores are summed to calculate summary scores for autonomous motivation, introjected regulation, external regulation, and amotivation, with higher scores indicating greater endorsement of motivation subtypes.

*History of weight loss attempts* was collected in 5 studies. All 4 clinical trials used the WALI [61], which asks respondents to report their age at the time of these efforts, weight loss method, and pounds lost. These occurrences will be summed to indicate participants’ history of weight loss attempts. NHANES collected these data by prompting participants to indicate if they attempted weight loss during the past year, and if so, asking whether they used specific weight loss methods. Participants’ responses to these prompts will be summed to indicate the total number of weight loss methods used during the past year.

**Table 3:** Measures of individual difference characteristics by study.

Characteristic	NHANES^a^	MIDUS^b^	ENACT	IMPACT	FitLink Pilot	FitLink Full
BMI	Measured	Measured	Measured	Measured	Measured	Measured
CVD^c^ risk	Individual questions (diagnosis)	Individual questions (diagnosis)	WALI^d^ and BMI	WALI and BMI	WALI and BMI	WALI and BMI
Depressive symptoms	PHQ-9^e^	CES-D^f^	BDI-II^g^	BDI-II	WALI	BDI-II
PA motivation	—^h^	—	TSRQ^i^	TSRQ	TSRQ	—
Weight loss attempts	Self-reported (past year)	—	WALI	WALI	WALI	WALI
Social comparison	—	Comparison of CVD risk	INCOM^j^	—	—	INCOM

^a^NHANES: National Health and Nutrition Examination Survey.

^b^MIDUS: Midlife in the United States.

^c^CVD: cardiovascular disease.

^d^WALI: Weight and Lifestyle Inventory.

^e^PHQ: Patient Health Questionnaire.

^f^CES-D: Center for Epidemiologic Studies Depression Scale.

^g^BDI: Beck Depression Inventory.

^h^Not applicable.

^i^TSRQ: Treatment Self-Regulation Questionnaire.

^j^INCOM: Iowa-Netherlands Comparison Orientation Measure.

**Table 4:** Cardiovascular disease (CVD) risk conditions assessed by the study.

CVD risk condition	NHANES^a^	MIDUS^b^	ENACT	IMPACT	FitLink Pilot	FitLink Full
Hypertension	✓	✓	✓	✓	✓	✓
High cholesterol	✓					
Prediabetes	✓					
Type 2 diabetes	✓	✓	✓	✓	✓	✓
Obesity (BMI ≥30 kg/m^2^)	✓	✓	✓	✓	✓	✓
Smoker	✓	✓	✓	✓	✓	

^a^NHANES: National Health and Nutrition Examination Survey.

^b^MIDUS: Midlife in the United States.

### Ethical Considerations

The coordinated, secondary analysis plan outlined here is approved as exempt (no human subjects enrollment) by the Institutional Review Board at Rowan University and Rowan-Virtua School of Osteopathic Medicine (protocol number PRO-2021-550). Original data collection for public datasets and clinical trials was approved by the organizations’ home institutional review boards, and participants received monetary compensation for their time and effort [1,52-56]. All consent processes involved authorization for secondary analyses. Datasets were anonymized or deidentified prior to analyses.

### General Analysis Plan

**Figure 1:**
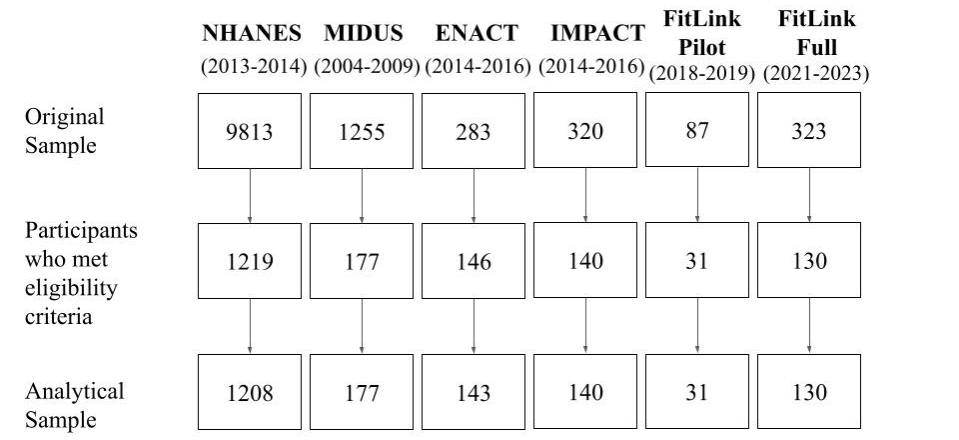
Anticipated analytical sample sizes for included studies. MIDUS: Midlife in the United States; NHANES: National Health and Nutrition Examination Survey.

Our primary analyses will examine how device-assessed PA behavior varies within persons across days of participation, using multilevel models. Each dataset includes summary indicators of PA on a given day, and days are nested within persons, creating dependence best accounted for by 2-level multilevel models. Primary outcomes of interest will be MIMS units, activity counts, and steps per day, as available in each dataset. As noted, data from participants who met demographic and medical criteria were included in the present analyses. We also set minimum PA monitoring device wear time to 10 hours of wake time per day, and days with <10 hours of valid wear time were excluded from analyses. The original, reduced, and analytical sample sizes for each study based on these criteria are provided in Figure 1.

### Coordinated Analysis Approach

As we have multiple datasets that share subsets of our outcomes and moderators of interest, we will use coordinated analysis to arrive at conclusions [50]. In coordinated analysis, models are parameterized and fit to datasets, and statistical results from specific datasets are compared and contrasted to efficiently replicate substantive conclusions [63,64]. Given the differences in study design, we will fit equivalent models separately across datasets, and standardized estimates can be generated to determine the consistency of effects with respect to direction and relative size. As in our previous work, we will use semipartial correlation coefficients as standardized effect size estimates [65-68]. Fitting separate models also allows for exploration of dataset-specific covariates and associations to address substantive questions relevant to individual datasets.

### Aim 1

To address our first aim, we will fit models that examine whether daily parameters of PA behavior at level 1 change within a person as a function of day of measurement to describe the pattern of measurement reactivity. For these models, we will examine day of measurement as a continuous predictor of PA using both linear and quadratic trends. Further, we have the opportunity to consider the day of measurement as a categorical predictor to identify whether a particular day of measurement shows greater or lower activity counts relative to adjacent days. As in previous work, we will document patterns where day 1 and days 1-2 meaningfully differ from other days of observation as measurement reactivity [29,37,65,69].

### Aim 2

We will then add moderators of change across days to address our second aim: to understand whether patterns of measurement reactivity differ based on BMI, percentage of CVD risk factors assessed, depressive symptoms, and gender. We will also examine whether patterns of measurement reactivity depend on motivation to engage in PA, social comparison responses, and previous attempts to lose weight. All moderators will be entered at level 2 as between-person predictors, and the cross-level interaction with the day of measurement will be included to assess for differences in patterns of measurement reactivity. We will also examine whether any reactivity that does emerge differs between genders. To do this, we will incorporate the 3-way interaction among gender, day of measurement, and the above moderators. Significant interactions will be probed with specific contrasts and visualizations separately for each group for categorical moderators or at 1 SD above and below the mean for continuous moderators.

### Aim 3

To address our third aim, we will use standardized estimates to compare the effect of the day of measurement on PA summary scores across study designs and device characteristics. Consistent with our follow-up analyses for our second aim, we will incorporate a cross-level interaction with gender and day of measurement to determine whether any effects of study design and device type depend on the gender of the individual being measured.

## Results

The datasets described provide 11,707 valid PA observations across 1832 adults in midlife with ≥1 risk factor for CVD. As indicated, participant characteristics for each study can be found in Table 2. Dataset acquisition is complete, and data cleaning in preparation for analysis is underway. We expect to complete analyses by April 2025 and to make full results available by July 2025.

## Discussion

This study will be the first to systematically investigate PA measurement reactivity in a population with elevated CVD risk using a coordinated multilevel analysis. Analyses will be executed on a large, diverse sample of midlife adults with elevated risk for CVD, who are frequent targets of PA promotion and who make up substantial subgroups in broader PA programs [2-7]. A critical advantage of the proposed analytic approach is that days will be treated separately and nested within individuals to determine the extent of potential effects of reactivity on estimates of averages [22]. This approach will also be applied across multiple research designs, PA monitoring devices, and individual difference characteristics, allowing for the identification of potential moderators of PA measurement reactivity responses.

A noteworthy limitation of our approach is that there is no opportunity to compare the same PA outcome across all studies. Only certain variables are publicly available for NHANES and MIDUS (ie, total activity counts and MIMS units, respectively), and these cannot be converted to estimates of steps or minutes of activity per day [70]. Conversely, only the latter are available for clinical trials, and these cannot be converted to corresponding units. To address this, we will report and compare standardized effect sizes across outcomes and studies [24]. In addition, a subset of participants in each study did not contribute enough valid PA data to be included, and participants who were included occasionally had days where valid data were missing due to insufficient wear time on that day (<20% of expected days) [25]. An additional advantage of a multilevel modeling approach is that these models are robust to missing data [71,72]. Thus, estimates and conclusions are unlikely to be affected by the observed level of missingness at the day level [25,28]. At the person level, we will also compare participants who were included in analyses to those who were excluded for having too few days of valid PA data. This will allow us to determine whether these groups differ with respect to demographic or medical characteristics and to what extent this level of missingness might impact the generalizability of our findings.

As noted, there is considerable debate about the need for substantial steps to address PA measurement reactivity, such as adding and removing days of observation or blinding participants to PA feedback from their measurement devices [25,29-31,73]. Limitations notwithstanding, findings from this study will indicate whether such steps are warranted among adults in midlife with elevated CVD risk and under what circumstances (eg, across participants or study designs vs for certain subgroups or study designs). Adults in this population are primary targets and recipients of PA assessment research and intervention, as evidence consistently shows low PA in this group despite considerable PA promotion efforts. However, very little PA measurement reactivity work has focused on this group. Drastic measures to minimize reactivity responses may improve PA estimates if reactivity is substantial; if not, such measures are likely to limit the benefits of self-monitoring in an intervention [74], as well as waste resources and increase participant burden for those who are most in need of support. Alternatively, there may be subgroups for whom or research contexts in which PA measurement reactivity is particularly problematic. Focusing attention on low-burden ways to minimize reactivity responses in situations where it is most likely to bias PA estimates would be more cost-effective than universal measures. For example, introducing participants to the concept and the possibility of reactivity, encouraging participants to be aware of it, and emphasizing engagement in normal PA behavior despite research participation or the presence of a monitor may effectively mitigate effects on PA estimates [65,75]. Such an approach, rather than adding days of assessment, would also increase the accessibility of research and intervention for midlife adults, who often have busy, unpredictable schedules [76] and who may find additional days of assessment overwhelming (and therefore, decline to participate or withdraw after enrollment [77,78]). Findings from this study will provide high-quality evidence to determine the effect of measurement reactivity on PA estimates in a key population of interest, and thereby contribute to best practice recommendations for measuring PA in daily life [31,38].

## Abbreviations

CES-D: Center for Epidemiologic Studies Depression Scale
CVD: cardiovascular disease
INCOM: Iowa–Netherlands Comparison Orientation Measure
MIDUS: Midlife in the United States Study
MIMS: monitor-independent measurement summary
MVPA: moderate-to-vigorous physical activity
NHANES: National Health and Nutrition Examination Survey
PA: physical activity
PHQ-9: Patient Health Questionnaire
TSRQ: Treatment Self-Regulation Questionnaire
WALI: Weight and Lifestyle Inventory
WELL: Weight Eating and Lifestyle

## Data Availability

Data from the National Health and Nutrition Examination Survey (NHANES) and the Midlife in the United States Study (MIDUS) are publicly available from the Inter-university Consortium for Political and Social Research. Data analyzed from the clinical trials described earlier are available from the corresponding author upon reasonable request.

## References

[1] Brim OG, Ryff CD, Kessler RC (2019). How Healthy Are We? A National Study of Well-Being at Midlife.

[2] Rodgers JL, Jones J, Bolleddu SI, Vanthenapalli S, Rodgers LE, Shah K, Karia K, Panguluri SK (2019). Cardiovascular risks associated with gender and aging. J Cardiovasc Dev Dis.

[3] Matthews KA, Crawford SL, Chae CU, Everson-Rose SA, Sowers MF, Sternfeld B, Sutton-Tyrrell K (2009). Are changes in cardiovascular disease risk factors in midlife women due to chronological aging or to the menopausal transition?. J Am Coll Cardiol.

[4] Appelman Y, van Rijn BB, Ten Haaf ME, Boersma E, Peters SAE (2015). Sex differences in cardiovascular risk factors and disease prevention. Atherosclerosis.

[5] Karvinen S, Jergenson MJ, Hyvärinen M, Aukee P, Tammelin T, Sipilä S, Kovanen V, Kujala UM, Laakkonen EK (2019). Menopausal status and physical activity are independently associated with cardiovascular risk factors of healthy middle-aged women: cross-sectional and longitudinal evidence. Front Endocrinol (Lausanne).

[6] Kapoor E, Collazo-Clavell ML, Faubion SS (2017). Weight gain in women at midlife: a concise review of the pathophysiology and strategies for management. Mayo Clin Proc.

[7] Piercy KL, Troiano RP (2018). Physical activity guidelines for Americans from the US Department of Health and Human Services. Circ Cardiovasc Qual Outcomes.

[8] Shiroma EJ, Lee I (2010). Physical activity and cardiovascular health: lessons learned from epidemiological studies across age, gender, and race/ethnicity. Circulation.

[9] (2019). National health expenditure data. Centers for Medicare & Medicaid Services.

[10] Waters LA, Galichet B, Owen N, Eakin E (2011). Who participates in physical activity intervention trials?. J Phys Act Health.

[11] Cooke R, Jones A (2017). Recruiting adult participants to physical activity intervention studies using sport: a systematic review. BMJ Open Sport Exerc Med.

[12] Murray JM, Brennan SF, French DP, Patterson CC, Kee F, Hunter RF (2017). Effectiveness of physical activity interventions in achieving behaviour change maintenance in young and middle aged adults: a systematic review and meta-analysis. Soc Sci Med.

[13] Hooker SP, Harmon B, Burroughs EL, Rheaume CE, Wilcox S (2011). Exploring the feasibility of a physical activity intervention for midlife African American men. Health Educ Res.

[14] Mellor D, Connaughton C, McCabe MP, Tatangelo G (2017). Better with age: a health promotion program for men at midlife. Psychol Men Masculinity.

[15] Ballon-Landa E, Parsons JK (2018). Nutrition, physical activity, and lifestyle factors in prostate cancer prevention. Curr Opin Urol.

[16] Griffith DM, Gunter K, Allen JO (2011). Male gender role strain as a barrier to African American men's physical activity. Health Educ Behav.

[17] Arigo D, Romano KA, Pasko K, Travers L, Ainsworth MC, Jackson DA, Brown MM (2022). A scoping review of behavior change techniques used to promote physical activity among women in midlife. Front Psychol.

[18] Grossman JA, Arigo D, Bachman JL (2018). Meaningful weight loss in obese postmenopausal women: a pilot study of high-intensity interval training and wearable technology. Menopause.

[19] Katzmarzyk PT, Lee I, Martin CK, Blair SN (2017). Epidemiology of physical activity and exercise training in the United States. Prog Cardiovasc Dis.

[20] Nomura T, Kawae T, Kataoka H, Ikeda Y (2018). Aging, physical activity, and diabetic complications related to loss of muscle strength in patients with type 2 diabetes. Phys Ther Res.

[21] Parvathaneni K, Surapaneni A, Ballew SH, Palta P, Rebholz CM, Selvin E, Coresh J, Grams ME (2021). Association between midlife physical activity and incident kidney disease: the atherosclerosis risk in communities (ARIC) study. Am J Kidney Dis.

[22] Steene-Johannessen J, Anderssen SA, van der Ploeg HP, Hendriksen IJM, Donnelly AE, Brage S, Ekelund U (2016). Are self-report measures able to define individuals as physically active or inactive?. Med Sci Sports Exerc.

[23] Jaeschke L, Steinbrecher A, Jeran S, Konigorski S, Pischon T (2018). Variability and reliability study of overall physical activity and activity intensity levels using 24 h-accelerometry-assessed data. BMC Public Health.

[24] Craig CL, Tudor-Locke C, Cragg S, Cameron C (2010). Process and treatment of pedometer data collection for youth: the Canadian physical activity levels among youth study. Med Sci Sports Exerc.

[25] Baumann S, Groß S, Voigt L, Ullrich A, Weymar F, Schwaneberg T, Dörr M, Meyer C, John U, Ulbricht S (2018). Pitfalls in accelerometer-based measurement of physical activity: the presence of reactivity in an adult population. Scand J Med Sci Sports.

[26] Scott JJ, Morgan PJ, Plotnikoff RC, Trost SG, Lubans DR (2014). Adolescent pedometer protocols: examining reactivity, tampering and participants' perceptions. J Sports Sci.

[27] French DP, Sutton S (2010). Reactivity of measurement in health psychology: how much of a problem is it? What can be done about it?. Br J Health Psychol.

[28] König LM, Allmeta A, Christlein N, Van Emmenis M, Sutton S (2022). A systematic review and meta-analysis of studies of reactivity to digital in-the-moment measurement of health behaviour. Health Psychol Rev.

[29] Zhu X, Haegele JA (2019). Reactivity to accelerometer measurement of children with visual impairments and their family members. Adapt Phys Activ Q.

[30] Motl RW, McAuley E, Dlugonski D (2012). Reactivity in baseline accelerometer data from a physical activity behavioral intervention. Health Psychol.

[31] Clemes SA, Deans NK (2012). Presence and duration of reactivity to pedometers in adults. Med Sci Sports Exerc.

[32] Conner T, Lehman B (2012). Getting started: launching a study in daily life. Handbook of Research Methods For Studying Daily Life.

[33] Davis R, Loprinzi P (2016). Examination of accelerometer reactivity among a population sample of children, adolescents, and adults. J Phys Act Health.

[34] Niemelä M, Kangas M, Farrahi V, Kiviniemi A, Leinonen A, Ahola R, Puukka K, Auvinen J, Korpelainen R, Jämsä T (2019). Intensity and temporal patterns of physical activity and cardiovascular disease risk in midlife. Prev Med.

[35] Vaccarezza M, Papa V, Milani D, Gonelli A, Secchiero P, Zauli G, Gemmati D, Tisato V (2020). Sex/gender-specific imbalance in CVD: could physical activity help to improve clinical outcome targeting CVD molecular mechanisms in women?. Int J Mol Sci.

[36] Klenk J, Peter RS, Rapp K, Dallmeier D, Rothenbacher D, Denkinger M, Büchele Gisela, Becker T, Böhm B, Scharffetter-Kochanek K, Stingl J, Koenig W, Riepe M, Peter R, Geiger H, Ludolph A, von Arnim C, Nagel G, Weinmayr G, Steinacker JM, Laszlo R (2019). Lazy Sundays: role of day of the week and reactivity on objectively measured physical activity in older people. Eur Rev Aging Phys Act.

[37] Hilgenkamp T, Van Wijck R, Evenhuis H (2012). Measuring physical activity with pedometers in older adults with intellectual disability: reactivity and number of days. Intellect Dev Disabil.

[38] French DP, Miles LM, Elbourne D, Farmer A, Gulliford M, Locock L, Sutton S, McCambridge J, MERIT Collaborative Group (2021). Reducing bias in trials due to reactions to measurement: experts produced recommendations informed by evidence. J Clin Epidemiol.

[39] König L M, Pasko K, Baga K, Harsora R, Arigo D (2025). Isolating the role of researcher observation on reactivity to the measurement of physical activity. Appl Psychol Health Well Being.

[40] Simon GE, Ludman EJ, Linde JA, Operskalski BH, Ichikawa L, Rohde P, Finch EA, Jeffery RW (2008). Association between obesity and depression in middle-aged women. Gen Hosp Psychiatry.

[41] Hansen BH, Holme I, Anderssen SA, Kolle E (2013). Patterns of objectively measured physical activity in normal weight, overweight, and obese individuals (20-85 years): a cross-sectional study. PLoS One.

[42] Lachman M, Lipsitz L, Lubben J, Castaneda-Sceppa C, Jette A (2018). When adults don't exercise: behavioral strategies to increase physical activity in sedentary middle-aged and older adults. Innov Aging.

[43] Gibbons FX, Buunk BP (1999). Individual differences in social comparison: development of a scale of social comparison orientation. J Pers Soc Psychol.

[44] Sreedhara M, Silfee V, Rosal M, Waring M, Lemon S (2018). Does provider advice to increase physical activity differ by activity level among US adults with cardiovascular disease risk factors?. Fam Pract.

[45] Hebert JR, Ma Y, Clemow L, Ockene IS, Saperia G, Stanek EJ, Merriam PA, Ockene JK (1997). Gender differences in social desirability and social approval bias in dietary self-report. Am J Epidemiol.

[46] Steinhardt MA, Dishman RK (1989). Reliability and validity of expected outcomes and barriers for habitual physical activity. J Occup Med.

[47] Barta WD, Tennen H, Litt MD (2012). Measurement reactivity in diary research. Handbook of Research Methods for Studying Daily Life.

[48] Fuller D, Colwell E, Low J, Orychock K, Tobin MA, Simango B, Buote R, Van Heerden D, Luan H, Cullen K, Slade L, Taylor NGA (2020). Reliability and validity of commercially available wearable devices for measuring steps, energy expenditure, and heart rate: systematic review. JMIR Mhealth Uhealth.

[49] Kanejima Y, Kitamura M, Izawa KP (2019). Self-monitoring to increase physical activity in patients with cardiovascular disease: a systematic review and meta-analysis. Aging Clin Exp Res.

[50] Hofer SM, Piccinin AM (2009). Integrative data analysis through coordination of measurement and analysis protocol across independent longitudinal studies. Psychol Methods.

[51] Jones C (2010). Archival data: advantages and disadvantages for research in psychology. Soc Personal Psychol Compass.

[52] NHANES questionnaires, datasets, and related documentation. Centers for Disease Control and Prevention.

[53] Butryn ML, Forman EM, Lowe MR, Gorin AA, Zhang F, Schaumberg K (2017). Efficacy of environmental and acceptance-based enhancements to behavioral weight loss treatment: the ENACT trial. Obesity (Silver Spring).

[54] Butryn ML, Godfrey KM, Call CC, Forman EM, Zhang F, Volpe SL (2021). Promotion of physical activity during weight loss maintenance: a randomized controlled trial. Health Psychol.

[55] Butryn ML, Martinelli MK, Crane NT, Godfrey K, Roberts SR, Zhang F, Forman EM (2020). Counselor surveillance of digital self-monitoring data: a pilot randomized controlled trial. Obesity (Silver Spring).

[56] Miller NA, Ehmann MM, Hagerman CJ, Forman EM, Arigo D, Spring B, LaFata EM, Zhang F, Milliron B, Butryn ML (2023). Sharing digital self-monitoring data with others to enhance long-term weight loss: a randomized controlled trial. Contemp Clin Trials.

[57] John D, Tang Q, Albinali F, Intille S (2019). An open-source monitor-independent movement summary for accelerometer data processing. J Meas Phys Behav.

[58] Kroenke K, Spitzer RL (2002). The PHQ-9: a new depression diagnostic and severity measure. Psychiatr Ann.

[59] Radloff LS (1977). The CES-D scale: a self-report depression scale for research in the general population. Appl Psychol Meas.

[60] Beck AT, Steer RA, Brown G (1996). Beck depression inventory-II (BDI-II)[Database record]. APA PsycTests.

[61] Wadden TA, Foster GD (2006). Weight and lifestyle inventory (WALI). Obesity (Silver Spring).

[62] Levesque CS, Williams GC, Elliot D, Pickering MA, Bodenhamer B, Finley PJ (2007). Validating the theoretical structure of the treatment self-regulation questionnaire (TSRQ) across three different health behaviors. Health Educ Res.

[63] Mogle J, Hill NL, Turner JR (2021). Individual differences and features of self-reported memory lapses as risk factors for alzheimer disease among adults aged 50 years and older: protocol for a coordinated analysis across two longitudinal data sets. JMIR Res Protoc.

[64] Hill NL, Bhargava S, Bratlee-Whitaker E, Turner JR, Brown MJ, Mogle J (2021). Longitudinal relationships between subjective cognitive decline and objective memory: depressive symptoms mediate between-person associations. J Alzheimers Dis.

[65] Arigo D, König LM (2024). Examining reactivity to the measurement of physical activity and sedentary behavior among women in midlife with elevated risk for cardiovascular disease. Psychol Health.

[66] Arigo D, Pasko K, Mogle JA (2020). Daily relations between social perceptions and physical activity among college women. Psychol Sport Exerc.

[67] Arigo D, Mogle JA, Smyth JM (2021). Relations between social comparisons and physical activity among women in midlife with elevated risk for cardiovascular disease: an ecological momentary assessment study. J Behav Med.

[68] Arigo D, Roberts SR, Butryn ML (2023). Social comparisons between group members during behavioural weight loss treatment: comparison direction, scale, and associations with weight loss maintenance. Psychol Health.

[69] Maher J, Arigo D, Baga K, Salvatore GM, Pasko K, Hudgins B, König LM (2024). Measurement reactivity in ecological momentary assessment studies of movement-related behaviors. J Meas Phys Behav.

[70] Lee IM, Moore CC, Evenson KR (2023). Maximizing the utility and comparability of accelerometer data from large-scale epidemiologic studies. J Meas Phys Behav.

[71] Hox J, Moerbeek M, Van de Schoot R (2017). Multilevel Analysis: Techniques and Application.

[72] Hoffman L (2015). Longitudinal Analysis: Modeling Within-Person Fluctuation and Change.

[73] French DP, Miles LM, Elbourne D, Farmer A, Gulliford M, Locock L, Sutton S, McCambridge J, MERIT Collaborative Group (2021). Reducing bias in trials from reactions to measurement: the MERIT study including developmental work and expert workshop. Health Technol Assess.

[74] Kanejima Y, Kitamura M, Izawa KP (2019). Self-monitoring to increase physical activity in patients with cardiovascular disease: a systematic review and meta-analysis. Aging Clin Exp Res.

[75] Arigo D, Brown MM, Pasko K, Ainsworth MC, Travers L, Gupta A, Downs DS, Smyth JM (2020). Rationale and design of the women's health and daily experiences project: protocol for an ecological momentary assessment study to identify real-time predictors of midlife women's physical activity. JMIR Res Protoc.

[76] Infurna FJ, Gerstorf D, Lachman ME (2020). Midlife in the 2020s: opportunities and challenges. Am Psychol.

[77] Anderson A, Getz K, Connor S, Getz N (2018). Trends in global public and patient perceptions of clinical research. Appl Clin Trials.

[78] Gabel M, Bollinger R, Knox M, Coble D, Grill J, Edwards D, Stark S, Lingler J (2022). Perceptions of research burden and retention among participants in ADRC cohorts. Alzheimer Dis Assoc Disord.

